# Self-Efficacy, Planning, or a Combination of Both? A Longitudinal Experimental Study Comparing Effects of Three Interventions on Adolescents’ Body Fat

**DOI:** 10.1371/journal.pone.0159125

**Published:** 2016-07-13

**Authors:** Aleksandra Luszczynska, Martin S. Hagger, Anna Banik, Karolina Horodyska, Nina Knoll, Urte Scholz

**Affiliations:** 1 Department of Psychology, SWPS University of Social Sciences and Humanities, Wroclaw, Poland; 2 Trauma, Health, & Hazards Center, University of Colorado at Colorado Springs, Colorado Springs, CO, United States of America; 3 Health Psychology and Behavioural Medicine Research Group, School of Psychology and Speech Pathology, Curtin University, Perth, Australia; 4 Faculty of Sport and Health Sciences, University of Jyväskylä, Jyväskylä, Finland; 5 Department of Education and Psychology, Freie Universität Berlin, Berlin, Germany; 6 Department of Psychology, University of Zurich, Zurich, Switzerland; University of Rome, ITALY

## Abstract

**Background:**

The superiority of an intervention combining two sets of theory-based behavior change techniques targeting planning and self-efficacy over an intervention targeting planning only or self-efficacy only has rarely been investigated.

**Purpose:**

We compared the influence of self-efficacy, planning, and self-efficacy+planning interventions with an education-based control condition on adolescents’ body fat, assuming mediating effects of respective social cognitive variables and moderate-to-vigorous physical activity (MVPA). The moderating role of the built environment was examined.

**Methods:**

Participants (*N* = 1217, aged 14–18 years) were randomly assigned to four conditions: planning (*n* = 270), self-efficacy (*n* = 311), self-efficacy+planning (*n* = 351), and control (*n* = 285). The measurement was conducted at baseline (T1), two-month follow-up (T2), and fourteen-month follow-up (T3). Interventions/control group procedures were delivered at T1 and T2. Percent of body fat tissue (measured at T1 and T3) was the main outcome. Social cognitive mediators (self-efficacy and planning) were assessed at T1 and T2. The behavioral mediator (MVPA) and the presence of built MVPA facilities (the moderator) were evaluated at T1 and T3.

**Results:**

Similar small increases of body fat were found across the three intervention groups, but the increment of body fat was significantly larger in the control group. On average, differences between control and intervention groups translated to approximately 1% of body fat. Effects of the interventions on body fat were mediated by relevant social cognitive variables and MVPA. A lower increase of body fat was found among intervention group participants who had access to newly-built MVPA facilities.

**Conclusions:**

We found no superiority of an intervention targeting two social cognitive variables over the intervention targeting one cognition only.

## Introduction

Prevention of overweight and body fat gain is among the key aims of interventions addressing physical activity or healthy nutrition among adolescents [1, 2]. However, the effects of behavior change interventions on obesity prevention are inconclusive if objective indicators of weight loss such as the body mass index are the main outcomes [3]. These inconclusive effects stem from the fact that changes in body mass index may poorly reflect dynamic changes in body composition taking place in adolescence [4]. In fact, using body mass index as an indicator of body composition may lead to severe classification errors because adolescents classified as overweight may have large bone and muscle mass [4]. Therefore, overweight prevention interventions for adolescents should target the changes of body composition and its major components, such as fat mass, as well as the behavioral mediators of changes in body composition, such as physical activity and diet change [2, 3].

There are well-documented associations between indicators of changes in body composition such as total body fat and behavioral mediators such as physical activity [2, 5]. In particular, moderate-to-vigorous physical activity (MVPA) predicts total body fat in a general population of adolescents, but the relationships are negligible for physical activity of light intensity [5]. Although body fat is expected to increase across adolescence, reviews of longitudinal research suggested that MVPA has a protective effect against a higher body fat gain [2]. Recognizing existing evidence for health benefits and prevention of excessive weight gain, the World Health Organization [1] recommended that adolescents should accumulate at least 60 min of MVPA per day.

### Self-Efficacy, Planning, and Physical Activity

Social cognitive theory is one of the approaches most frequently used as a basis for interventions targeting obesity prevention and PA among adolescents [3]. In particular, self-efficacy as a key predictor within social cognitive theory has been shown to be a proximal, consistent, and strong predictor of variance in PA and interventions based on the construct have been shown to be effective [6, 7]. Self-efficacy is defined as beliefs about the ability to act upon one’s intention to engage in a particular behavior (e.g., participation in MVPA), regardless of barriers (e.g., feeling tired; [6]). Those beliefs are directly related to the maintenance of PA and relapse prevention [6]. Research has shown that self-efficacy is one of the most consistent predictors of a smaller decline in MVPA across adolescence [8]. Systematic reviews of self-efficacy interventions for adults suggest small to moderate effects on PA [9]. However, long-term effects of brief self-efficacy interventions on adolescents’ PA (including MVPA) are inconclusive [3, 10].

Implementation intentions or planning [11] is another theoretical approach focusing on mechanisms responsible for the attainment of behavioral goal in health contexts including PA [12]. The action planning approach assumes that cues-to-action should make reference not only to ‘when’ and ‘where’ the behavior should be enacted, but also to ‘how’ it should be done [13], whereas coping plans focus on how the behavior should be done if barriers arose [14]. Forming action and coping plans is related to PA in correlational and experimental studies conducted among adults [15, 16]. Much less is known about the influence of planning interventions on adolescents’ PA.

In sum, self-efficacy and planning are the key ‘building blocks’, often used in interventions addressing health behaviors [13]. Longitudinal correlational studies have provided evidence for the assumption that self-efficacy and planning operate in concert when predicting PA [17]. However, to our knowledge there is no evidence that a combination of self-efficacy and planning would result in substantially larger changes in MVPA, compared to interventions targeting self-efficacy only or planning alone [13]. The current study aims to address this gap by developing an intervention assessing the main and interactive effects of intervention techniques based on these constructs in a factorial design.

### Mechanisms Explaining Effects of Self-Efficacy and Planning Interventions

Planning and self-efficacy interventions may operate through specific mechanisms. For example, effects of self-efficacy interventions were mediated by a change in self-efficacy but not mediated by a change in the use of planning [18]. The evaluation of underlying mechanisms may be achieved using a mediation analysis, testing whether assignment to the intervention condition explains change in MVPA indirectly, through a change in the psychological variables matched to the intervention’s behavior change techniques. Unfortunately, research on behavior change interventions rarely provides explicit tests of the underlying mediating mechanisms. Analyses of the effects of any theory-based psychological mediators explaining the effects of interventions on weight-related outcomes and PA measured at long-term were conducted in less than 25% of trials accounting for the mediators [19]. Without specifying and testing for the underlying mechanisms, even a well-designed study cannot be informative of *how* an intervention worked [20].

We identified only four studies comparing the effects of planning and self-efficacy interventions, with two testing the mediating role of social cognitive variables. Compared to a self-efficacy intervention, a planning intervention turned out to be more effective in determining long-term changes in adolescent smoking [21]. The study did not test the potential cognitive mediators. Luszczynska et al. [18] found that both self-efficacy and planning interventions affected fruit and vegetable intake at 12-month follow-up, but energy-dense food intake changed only in the self-efficacy group. Respective social-cognitive variables mediated the effects on behavior. Guillaumie, Godin, Manderscheid, Spitz, and Muller [22] found that effects of three interventions (planning, self-efficacy, and a combination of planning and self-efficacy) on fruit and vegetable intake, evaluated at the 3-month follow-up, were mediated by planning but not by self-efficacy. Guillaumie, Godin, Maderscheid, Spitz, and Muller [23] also showed that the same three interventions influenced fruit/vegetable intake at 6-month follow-up, but not at 12-month follow-up.

### Combining Intervention Components

Although there is a large body of evidence for the effectiveness of multi-component interventions which use multiple sessions [24], it remains unclear which of the components are responsible for behavior change and whether there is any redundancy across the components. Furthermore, it is unknown whether combining two effective components would result in a substantially larger effect, over and above single-component interventions. The evaluation of superiority of a multi-component intervention over one-component intervention should be conducted controlling for the forms of delivery. Among delivery aspects, the length of the intervention is consistently related with weight-related and behavioral outcomes such as PA [3]. Thus, the conclusive evidence may be obtained if the interventions targeting single or multiple social cognitive variables use a similar delivery protocol and have the same duration.

So far, only two studies [22, 23] compared the effects of self-efficacy, planning, and combined self-efficacy + planning interventions. The studies are not conclusive regarding the mediating mechanisms and applied only self-report measurement of outcomes. Thus, the effects and underlying mechanisms of the single-component and combined interventions require further and more thorough testing.

### The Effects of Built Environment, Providing Opportunities for MVPA

Besides the effects of social cognitive variables on behavior-related outcomes, social-cognitive theory [25] highlights the role of environmental factors. Social cognitive variables, behaviors, and environment are in continuous and reciprocal relationships and all three of them have to be accounted for to fully explain individual’s functioning [25]. Systematic reviews indicated that the built environment is among the predictors of excessive weight gain indicators in adolescence [26]. In particular, built facilities providing opportunities for PA in close proximity to the school environment have a positive effect on adolescents’ MVPA [27]. Built PA facilities are associated with the greatest odds of engaging in frequent bouts of MVPA [28]. The evidence for the interaction effects of self-efficacy (or control-related social cognitive variables) and built PA facilities on PA is inconclusive [29]. Interaction effects of built environment and planning intervention on PA were not analyzed. However, research has indicated that psychosocial interventions focused on PA and dietary change among adolescents were more likely to produce significant effects if combined with changes in built environment, in particular, built PA facilities [3]. Thus, the presence of such facilities may enhance the effects of self-efficacy or planning interventions.

### The Present Study

This longitudinal experimental study tested the effects of three brief interventions, each using a single or combination of sets of behavior change techniques to change theory-based social cognitive variables: (1) prompting the formation of plans, (2) prompting self-efficacy beliefs, and (3) prompting planning + self-efficacy relative to an active ‘education only’ control group. The intervention and control group procedures targeted an increase of MVPA and a concomitant improvements in body composition in adolescents aged 14–18 years. It was hypothesized that participants assigned to the interventions would exhibit a smaller increase in body fat at 14-month follow-up compared to controls. We also investigated whether the combined planning + self-efficacy intervention would have larger effects on the main outcome (body fat) than single-component interventions. Second, it was hypothesized that the effects of the intervention conditions on body fat at the 14-month follow-up would be mediated by their respective social cognitive and behavioral constructs: self-efficacy and planning at T2 (Mediator 1), and by MVPA at T3 (Mediator 2). We expected that the effects of the interventions including the planning component (i.e., planning intervention and self-efficacy + planning intervention) would be mediated by planning, whereas the effects of the interventions including the self-efficacy component (i.e., self-efficacy intervention and self-efficacy + planning intervention) would be mediated by self-efficacy. Finally, we explored whether the effects of the intervention (both direct and indirect effects, via their respective psychological variables and MVPA) on body fat would be moderated by a specific component of the built environment: the presence of built PA facilities located in close proximity of schools.

## Materials and Methods

### Participants

**Fig 1:**
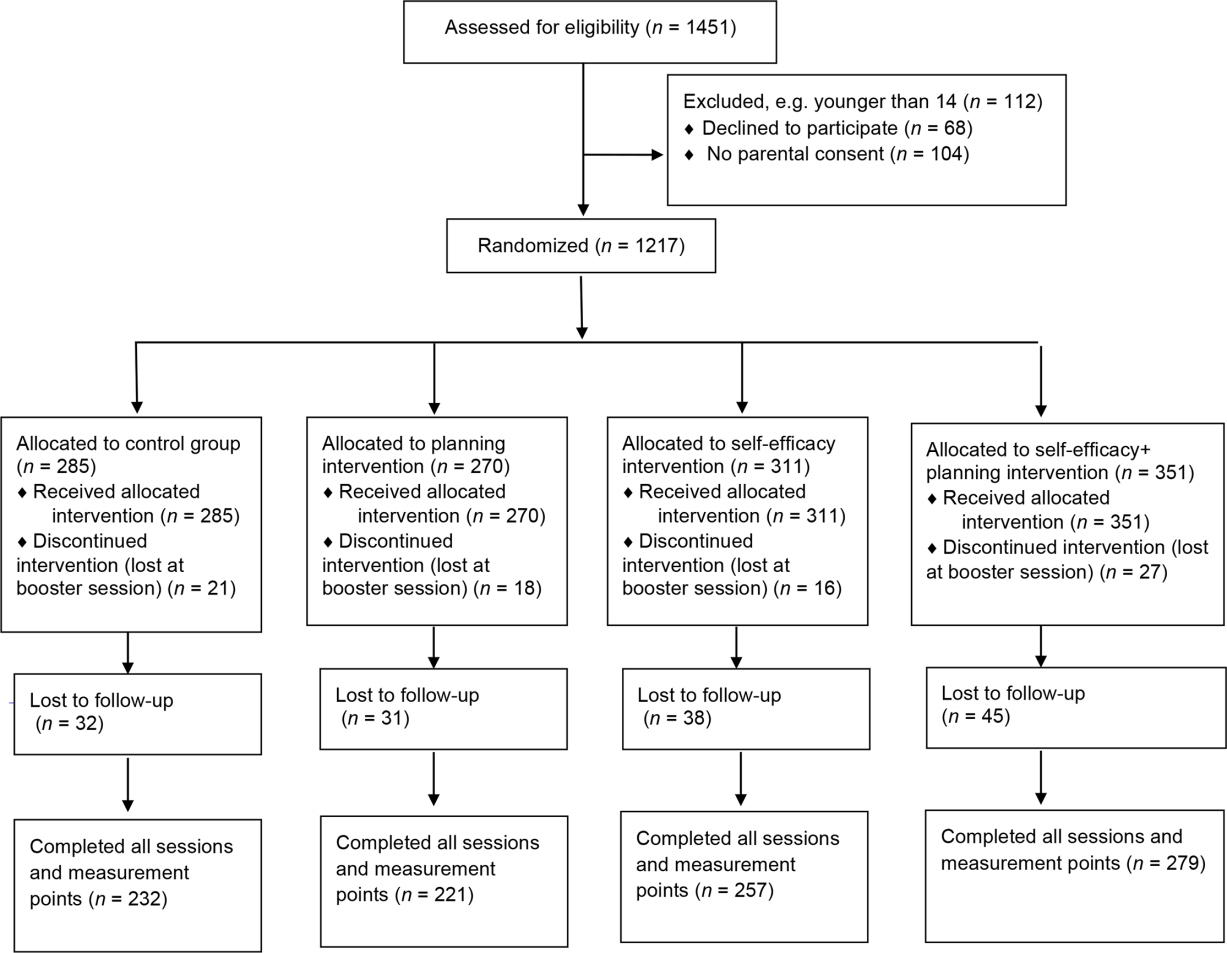
The flowchart.

Participants were adolescents (*N* = 1217; *M* age = 16.45, *SD* = 0.70, age range 14–18 years), 58% were girls, and 98% were of White ethnicity. Using BMI cut-off scores for overweight or obesity, adjusted for age and gender [30] it was determined that 19% (*n* = 230) were overweight or obese at T1 and only nine adolescents were underweight. BMI ranged from 16.10 to 38.78 (*M* = 21.87, *SD* = 3.22) at T1 and from 16.14 to 41.26 (*M* = 22.03; *SD* = 3.43) at T3. The majority of participants (*n* = 988; 81.3%) remained in the study across all three data collection points. Participant flow is presented in Fig 1. Adolescents younger than 14 years old and those who declared that they would change schools during the following year (e.g., due to graduation or moving to another region) were excluded. No other exclusion criteria were applied. Potential respondents were recruited during the classes.

Parental written consent was obtained prior to data collection. Parents attending annual meetings at schools were informed about the purpose of the study and if they agreed to their child’s participation they were asked to sign the consent form with their own and their child’s name. Informed written consent was also obtained from all adolescents included in the study. All students received information about the study aims and the procedures. In particular, the study was introduced as promoting moderate and vigorous physical activity and healthy body weight and composition. The aim referring to the change of body fat was not disclosed. Participants who were concerned or distressed by these issues were invited to contact the experimenters after the respective wave of data collection (i.e., a consultation with a clinical psychologist was available). Those who agreed and provided informed consent were assigned personal codes to secure anonymity and identification.

Participants were randomly assigned to the active control education group (*n* = 285; 23.4%), the planning group (*n* = 270; 22.2%), the self-efficacy group (*n* = 311; 25.5%), or the self-efficacy + planning group (*n* = 351; 28.9%). The sequence was created by an external researcher with a random digit generator. The generated sequence of digits was applied to the order of participants entering each classroom at T1, starting in each new class from the first generated number (for similar procedures see [18]). This procedure was chosen to facilitate the data collection taking place concurrently in several locations. However, it relied on repeating the first 50 digits of the generated sequence in each classroom, and therefore resulted in unequal group sizes.

### Measures

Descriptive statistics for all variables are presented in Table 1.

**Table 1:** Correlations between study variables and descriptive statistics for the total sample (*N* = 1217).

	Variable	2	3	4	5	6	7	8	9	10	11	12	*M* (*SD*)
1	MVPA (T1)	.45	.04	.06	.07	.06	.10	.06	.09	-.01	-.02	.06	106.71 (62.50)
2	MVPA (T3)		.03	.04	.05	.05	.25	.03	.14	-.03	-.02	.04	108.34 (58.39)
3	Body fat (T1)			.76	-.11	-.03	-.06	-.03	-.12	.55	-.04	.57	21.64 (7.01)
4	Body fat (T3)				-.06	-.01	-.14	-.02	-.08	.52	-.02	.49	22.56 (6.50)
5	Intention (T1)					.15	.23	.20	.24	.01	.01	-.08	4.68 (1.35)
6	Self-efficacy (T1)						.48	.36	.03	-.01	.02	-.01	2.63 (0.93)
7	Self-efficacy (T2)							.05	.52	-.02	.03	-.05	2.78 (0.78)
8	Use of planning (T1)								.17	-.02	.02	-.02	2.68 (0.86)
9	Use of planning (T2)									.02	.05	-.05	2.72 (0.90)
10	BMI (T1)										.08	-.10	21.87 (3.21)
11	Age (T1)											.03	16.44 (0.70)
12	Gender												

All coefficients above .06 (or below -.06) are significant at p < .05, coefficients above .08 (or below -.08) are significant at p < .01 and coefficients above .10 (or below -.10) are significant at p < .001. MVPA–moderate-to-vigorous physical activity. Means for MVPA represent the total number of minutes per week. For other self-report measurements M and SD were calculated as mean item response. Gender: 1 = boys, 2 = girls; T1 –Time 1, the baseline; T2 –Time 2, 2 months after the baseline; T3 –Time 3, 14-month follow-up.

The main outcome, total body fat (T1 and T3) was measured with bioimpedance (BIA) method [31], which determines the electrical impedance of an electric current through body tissues. Fat tissue was estimated with Schaefer equation for BIA which is considered a reliable index of body fat in adolescent from primarily white backgrounds [32]. BIA scale (BF-100 and BF-25; Beurer) with a measurement error of 1% was used. Body weight and height (T1 and T3) were measured with standard medically approved telescopic height measuring rods and floor scales (models BF-100 or BF-25).

Moderate and vigorous physical activity (T1 and T3) was assessed using two items from Godin and Shephard’s [33] Leisure-Time Exercise Questionnaire (e.g., ‘Considering a 7-day period [a week], how many times on the average do you do the following kinds of exercise for more than 15 minutes during your free time: strenuous exercise [heart beats rapidly], e.g., running, jogging, hockey, soccer, basketball, cross-country skiing, vigorous swimming, vigorous long distance bicycling; moderate exercise [not exhausting], e.g., fast walking, tennis, easy bicycling, volleyball, badminton, easy swimming, alpine skiing, popular and folk dancing’). The two items were moderately correlated (T1: *r* = 0.47; T3: *r* = 0.44).

Use of physical activity planning (T1 and T2) was measured using four items (e.g., ‘I have my own plan regarding when to engage in exercise of moderate-to-vigorous intensity’; [17]). Responses ranged from 1 (‘definitely not’) to 4 (‘exactly true’). Cronbach’s α coefficients for this scale were .71 (T1) and .69 (T2).

Physical activity self-efficacy (T1 and T2) was measured using 9 items (e.g., ‘I am able to maintain regular MVPA even if I have to reorganize my daily life’; [16]). The responses ranged from 1 (‘definitely not’) to 4 (‘exactly true’). Cronbach’s α coefficients for this scale were .77 (T1) and .82 (T2).

MVPA intention (T1) was measured with two items (‘within the next month I intend to engage in MVPA on a regular basis’ and ‘within the next month, do you intend to follow the MVPA recommendations? [17]). Responses ranged from 1 (‘definitely not’) to 7 (‘exactly true’). The two items were correlated (*r* = .42).

Data referring to built PA facilities were collected at T1 and T3 in interviews with two teachers in each school (the year when the facility was built, its type, capacity, access, opening hours, adult supervision). We searched Google maps and governmental websites (e.g. https://www.orlik2012.pl/index.php/mapa) to identify whether facilities providing MVPA opportunities were available within a 1 km radius of the participants’ schools. The presence of respective facilities for MVPA was coded as 1 (‘present’) or 0 (‘absent’).

### Procedures

There were three measurement points: before the experimental procedures (T1), before the booster session (T2), and one year after the completion of the booster session (T3). The booster for experimental and control groups and T2 measurement took place at *M* = 2.11 months after T1 and T3 follow-up took place at *M* = 12.21 months after T2. The description of the protocol, the experimenters delivering the intervention, the intervention format, and the experimental materials used in the experimental groups and the control group is made available in S1 File (https://goo.gl/Yz6rSV). There were no changes to the intervention and control group protocol and procedures or deviations from the planned protocol. Data were collected between 2009–2015 from 14 public middle and high schools in rural (five schools, 36% of participants) and urban areas (nine schools, 64% of participants), which is similar to the rural and urban distribution of inhabitants in Poland (32% and 68%, respectively; [34]). The schools were located in the regions of representing lower economic development (four schools from the region with GDP of 68.5% of Polish GDP per capita), medium economic development (six schools from the region with GDP of 103.4%) and higher economic development (four schools from the region with GDP of 151.6%; [34]).

To test the role of the built environment we used a natural experiment design. During years 2008–2012 Poland was implementing a nation-wide policy, ‘Eagle 2012: My Playing Field’ which resulted in building 2700 pitches/team sports facilities near schools, accessible to students for free from 07:00 till 22:00. This nation-wide policy assumed that the number of these newly built facilities would be equal across administrative regions. Thus, the facilities were built across regions varying in urbanization and economic development. Seven schools (50%) had built PA facilities, defined as an outdoor built PA facility for soccer, basketball, and volleyball, with respective equipment and facilities (nets, locker rooms, lighting, fencing, adult supervision). The remaining schools (50%, *k* = 7) had no similar built facilities for MVPA available within a 1km radius of the participants’ schools.

Self-efficacy, planning, and self-efficacy + planning groups adopted specific sets of behavior change techniques designed to evoke change in the relevant social cognitive variables derived from theory and found to be related to health behavior in formative research [35]. Two independent researchers (one who had completed online training in behavior change techniques offered by University College London) evaluated the protocols for their content in terms of the behavior change techniques included in the intervention conditions [35]. The agreement coefficients (Cohen’s κs) were ≥ .61, *p* < .01. The following techniques [35] were included in the planning intervention protocol: action planning (7), barrier identification (8), prompting self-talk (33), relapse prevention/coping planning (35). The self-efficacy protocol included barrier identification (8), prompting focus on past success (18), and prompting self-talk (33). Applications of all techniques included references to planning or self-efficacy relevant to the experimental condition. The active control education group protocol did not include any of the behavior change techniques [35] applied in the experimental groups. The experimenters were instructed to avoid using other behavior change techniques that might interfere or augment those used in the assigned condition.

### Data Collection Procedures

At T1 questionnaires and the first part of the intervention materials were completed in classrooms. Biometric measures (body weight, height, and fat) were taken by the experimenters in the school nurse offices during the same day when the face-to-face component was delivered. After the biometric measurements, each student participated in the individual face-to-face component of the experimental procedures (see S1 File at https://goo.gl/Yz6rSV). At T2 (2-month follow-up) the experimenters returned to the schools and conducted the booster session (respective assessment, the group and face-to-face components). At T2 and T3 the experimenters returned to school for 3–5 times across 21 days to retrieve absent students.

The study was approved in 2008 by the Ethics Committee for Research in Human at SWPS University of Social Sciences and Humanities, Warsaw, Poland. At the time of receiving the approval, the respective ethics committee was not suggesting a public registration of the study. After recruitment the trial was registered with ClinicalTrials.gov (# NCT02689973; available at: https://goo.gl/RJOLgF).

Participants were blinded to group allocation. Experimenters were naïve to the purpose of the different types of intervention. They were informed that they would deliver four types of interventions aimed at nutrition and MVPA change. After initial training and after the study completion the experimenters were asked if they expected that the four types of forms might have different effects on adolescents’ MVPA. None of the experimenters indicated that they expected different effects of four experimental conditions on MVPA.

### Data Analysis

Repeated measures analysis of variance was used to investigate the effects of the group assignment on body fat. Intention to exercise, age, and gender were entered as covariates. There were no changes to the main outcome after the commencement of the trial.

**Fig 2:**
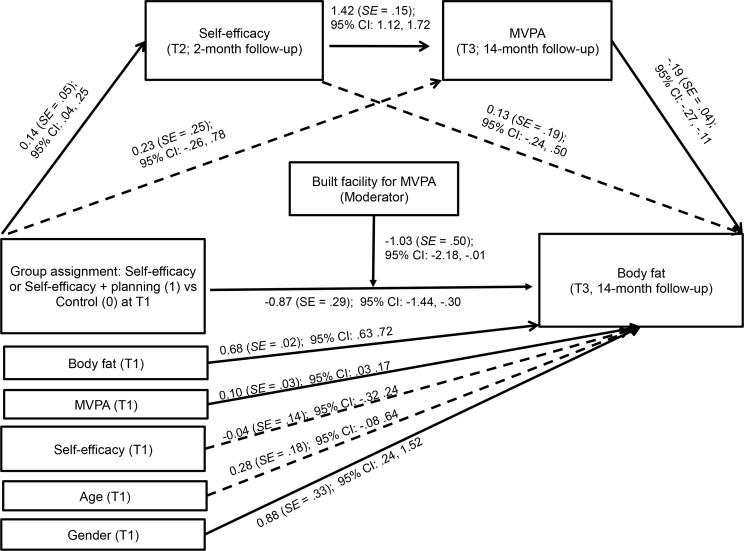
Effects of the group assignment (self-efficacy or self-efficacy + planning versus control) on body fat (14-month follow-up) mediated by self-efficacy and moderate-to-vigorous physical activity. Solid lines represent significant associations. MVPA–moderate-to-vigorous physical activity.

**Fig 3:**
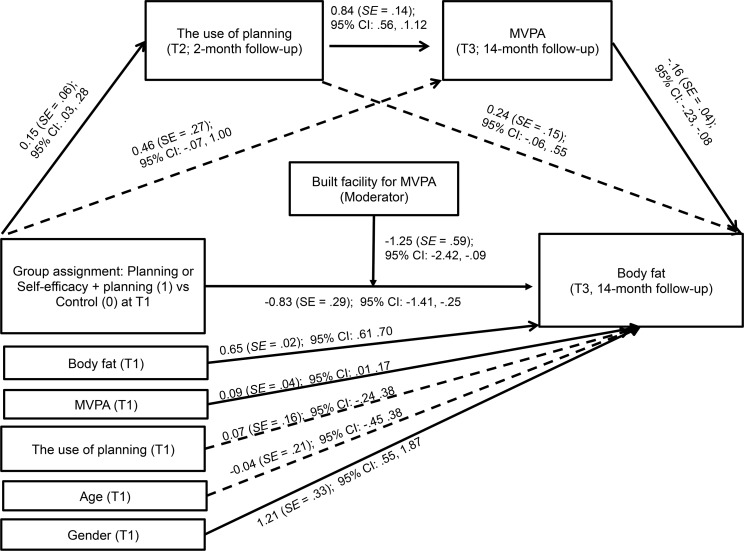
Effects of the group assignment (planning or self-efficacy + planning versus control) on body fat (14-month follow-up) mediated by planning and moderate-to-vigorous physical activity. Solid lines represent significant associations. MVPA–moderate-to-vigorous physical activity.

To test whether the effects of the behavior change techniques in each intervention group were mediated by their respective social cognitive variables, we performed sequential mediation analyses using the PROCESS program with 10,000 bootstrapped replications [36]. PROCESS permits the conduct of multiple mediator analysis in linear multiple regression models while accounting for covariates (T1 MVPA, T1 body fat, T1 mediators, T1 intention, age, and gender). We conducted multiple mediation analyses with sequential mediators (social cognitive variables at T2 as mediators in the first sequence; MVPA at T3 as mediators in the second sequence). Results of analyses are presented using two types of coefficients. A regression coefficient for each parameter is provided (see Fig 2 and Fig 3). Furthermore, PROCESS estimates the indirect effect coefficient (*θ*) for each indirect pathway (through respective mediators) between the independent variable (the group assignment) and the dependent variable (body fat at T3). The independent variables were coded as 1 (for self-efficacy as the mediator: the self-efficacy group and the self-efficacy + planning group; for planning as the mediator: the planning group and the self-efficacy + planning group) or 0 (the control group). Moderated mediation was conducted with PROCESS to test whether the indirect and direct effects observed in the mediation models were moderated by built PA facility (coded as 1 or 0). We calculated that to obtain effects of small size and retain power of .80 in multivariate analyses each group should have at least 260 participants.

Missing data analysis indicated that the data were missing completely at random (MCAR), with Little’s MCAR χ^2^ (637) = 348.68, *p* = 1.000. To follow the assumptions of intention-to-treat analysis missing data were treated with a maximum likelihood regression imputation with AMOS. Drop-out data were imputed with a maximum likelihood regression method and the analyses were conducted for the initial sample of 1217 participants.

## Results

### Preliminary Analyses

Randomization check indicated that at T1 respondents assigned to the four groups did not differ on key variables: gender, χ^2^ (4, 1217) = 10.89, *p* = .539, age, *F*(3, 1213) = 1.39, *p* = .243, BMI, *F*(3, 1213) = 1.14, *p* = .333, body fat tissue, *F*(3, 1213) = 0.86, *p* = .462, MVPA, *F* (3, 1213) = 1.45, *p* = .330, self-efficacy, *F*(3, 1213) = 1.08, *p* = .356, planning, *F*(3, 1213) = 0.77, *p* = .512, or physical activity intention, *F*(3, 1213) = 2.25, *p* = .081.

Attrition analysis indicated that drop-outs did not differ from completers on key study variables: gender, χ^2^ (1, 1217) = 2.91 *p* = .088, age, *F*(1, 1215) = 0.91, *p* = .341, BMI, *F*(1, 1215) = 0.72, *p* = .396, fat tissue, *F*(1, 1215) = 0.45, *p* = .504, MVPA, *F*(1, 1215) = 0.77, *p* = .380, self-efficacy, *F*(1, 1215) = 0.73, *p* = .393, planning, *F*(1, 1215) = 0.55, *p* = .457, or physical activity intention, *F*(1, 1215) = 0.34, *p* = .559. Participants in the two types of schools (with or without newly built MVPA facilities) differed at T1 in MVPA, with those from schools with MVPA facilities performing MVPA more often (*M* = 110.10, *SD* = 61.20) compared to students from remaining schools (*M* = 102.01, *SD* = 64.03), *F*(1, 1215) = 4.89, *p* = 0.026, η^2^ = .010. There were no significant differences in other T1 variables, all *p*s > .062.

Results of correlation analyses for the total sample (*N* = 1217) are displayed in Table 1. Higher levels of MVPA at T1 and T3 were related to intention, self-efficacy, and greater frequency of use of planning at T1 and T3, but the associations were weak. Lower levels of body fat (T1 and T3) were related to higher levels of self-efficacy and planning (T2).

### Intervention Effects on Body Fat

Repeated measures analysis of variance was conducted to test the effects of the group assignment on body fat (T1 and T3), with age, gender, and intention entered as covariates. There was a significant effect of time, *F*(1, 1210) = 3.923, *p* = .048, and a significant time x group interaction effect, *F*(3, 1210) = 3.09, *p* = .026, η^2^ = .008. There were significant effects of two covariates (gender: *F*[1, 1210] = 37.93, *p* < .001, η^2^ = .030; intention: *F*[1, 1210] = 5.16, *p =* .023, η^2^ = .004), but there was no effect of age (*p* = .223).

Pairwise comparisons revealed significant time x group interactions for the following comparisons: the self-efficacy vs control group, *F*(1, 591) = 4.34, *p* = .038, η^2^ = .007, the planning vs control group, *F*(1, 550) = 5.49, *p* = .019, η^2^ = .010, the self-efficacy + planning versus control group, *F*(1, 631) = 8.24, *p* = .004, η^2^ = .013. There were no time x group interactions for the following comparisons: self-efficacy vs self-efficacy + planning, *F*(1, 657) = 0.40, *p* = .526, η^2^ = .001, planning vs self-efficacy + planning, *F*(1, 616) = 0.08, *p* = .778, η^2^ < .001, and self-efficacy vs planning, *F*(1, 576) = 0.14, *p* = .713, η^2^ < .001. Across the two-group comparisons, effects of time, gender and intention were significant (all *p*s < .048). The inspection of means (Table 2) indicates that between T1 and T3 a small increase of body fat was found in the three intervention groups (*d*s from .08 to .12), but the increase in the control group was larger (*d* = 0.26).

**Table 2:** Descriptive statistics in the four study groups: moderate-to-vigorous physical activity, percent of body fat, self-efficacy beliefs and use of planning.

		Body fat (%)				Self-efficacy		The use of planning		Moderate-to-vigorous physical activity (minutes per week)			
	T1: *M* (*SD*)	T3: *M* (*SD*)	Average change in % of body fat from T1 to T3	Cohen’s *d*: T1 vs T3	Cohen’s *d*: experimental condition vs control	T1: *M* (*SD*)	T2: *M* (*SD*)	T1: *M* (*SD*)	T2: *M* (*SD*)	T1: *M* (*SD*)	T3: *M* (*SD*)	Cohen’s *d*: T1 vs T3	Cohen’s *d*: experimental condition vs control
Self-efficacy group (*n* = 311)	21.12 (7.28)	21.93 (6.74)	0.80	0.12	-0.23	2.56 (0.90)	2.86 (0.72)	2.68 (0.87)	2.58 (0.90)	106.38 (68.34)	108.64 (58.49)	0.04	0.11
Planning group (*n* = 270)	21.99 (6.66)	22.68 (6.32)	0.69	0.11	-0.12	2.68 (0.91)	2.81 (0.76)	2.64 (0.86)	2.87 (0.89)	102.09 (58.15)	112.85 (66.06)	0.17	0.17
Self-efficacy + planning group (*n* = 351)	21.69 (7.16)	22.33 (6.75)	0.64	0.08	-0.19	2.60 (0.95)	2.82 (0.76)	2.72 (0.85)	2.85 (0.88)	106.64 (59.83)	110.22 (51.62)	0.06	0.14
Control group (*n* = 285)	21.81 (6.87)	23.41 (5.87)	1.60	0.26		2.66 (0.91)	2.65 (0.75)	2.66 (0.86)	2.65 (0.90)	111.53 (62.01)	102.52 (58.21)	-0.15	

T1 –Time 1, the baseline; T2 –Time 2, 2-month follow-up; before booster session, 2 months after T1; T3 –Time 3, follow-up at 14 months after T1.

Additional analyses were conducted to test if the effects of the group assignment on body fat were moderated by participants’ overweight status at T1. Individuals with normal body weight (*n* = 977) and those with overweight or obesity (*n* = 230) were included, participants classified as underweight (*n* = 9) were excluded. There was no time x group x overweight status interaction, *F*(3, 1196) = 0.13, *p* = .943, η^2^ < .001. The pairwise comparisons between each of the experimental groups and the control group did not yield any significant time x group x overweight status interactions, all *p*s > .533. Furthermore, secondary analysis of the effect of the group assignment on change in body fat was conducted after excluding nine participants classified as underweight. Findings were similar to those observed for the total sample, with a significant time x group interaction, *F*(3, 1201) = 3.23, *p* = .022, η^2^ = .008, controlling for age, gender, and T1 intention.

### Intervention Effects on Social Cognitive Variables and MVPA

A series of repeated measures analyses of variance was conducted to test the effects of the group assignment on self-efficacy and the use of planning (T1 and T2), controlling for age, gender, and intention. For self-efficacy, there was no significant effect of time, *F*(1, 1210) = 0.62, *p* = .621, η^2^ < .001, but there was a time x group interaction, *F*(3, 1210) = 3.20, *p* = .023, η^2^ = .008. For the use of planning, there was no significant effect of time, *F*(1, 1210) = 0.60, *p* = .439, η^2^ < .001, but there was a time x group interaction, *F*(3, 1210) = 2.97, *p* = .031, η^2^ = .007.

Repeated measures analysis of variance was conducted to test the effects of the group assignment on MVPA at T1 and T3, with age, gender and intention entered as covariates. There was no effect of time, *F*(1, 1210) = 0.02, *p* = .881, but there was a significant overall time x group interaction effect, *F*(3, 1210) = 4.83, *p* = .023, η^2^ = .012. The effects of the three covariates were not significant (all *p*s > .324).

Next, we conducted pairwise comparisons of the effects of the four study groups to probe the significant time x group interaction effect. We found significant time x group interaction effects for the following comparisons: self-efficacy vs control group, *F*(1, 591) = 3.89, *p* = .049, η^2^ = .006, planning vs control group, *F*(1, 550) = 12.21, *p* < .001, η^2^ = .023, self-efficacy + planning vs control group, *F*(1, 631) = 7.54, *p* = .006, η^2^ = .012. However, there were no time x group interactions for the following comparisons: self-efficacy vs self-efficacy + planning, *F*(1, 657) = 0.29, *p* = .588, η^2^ < .001, planning vs self-efficacy + planning, *F*(1, 616) = 2.54, *p* = .112, η^2^ = .004, and self-efficacy vs planning, *F*(1, 576) = 2.81, *p* = .094, η^2^ = .005. Across the two-group comparisons, effects of time and covariates were not significant (all *p*s > .096). As indicated in Table 2, MVPA decreased over time in the control group (*d* = -0.15) whereas small increases of MVPA were observed in three intervention groups (*d*s from 0.04 to 0.17).

### The Mediating Effects of Social Cognitive Variables and MVPA

The first sequential mediation model (Fig 2) was designed to verify the mediating effects of self-efficacy (T2) and MVPA (T3) in the relationship between the group assignment (T1) and adolescents’ body fat (T3). The analyses were conducted with two study groups including the self-efficacy intervention component (i.e., the self-efficacy group and the self-efficacy + planning group) coded as 1 and the control group coded as 0 (*n* = 946). We controlled for MVPA (T1), body fat (T1), self-efficacy (T1), age, and gender. Results revealed that the association between the group assignment and body fat (T3) was mediated by self-efficacy (T2) and MVPA (T3) as indicated by a significant indirect effect (θ = -0.04, *SE* = 0.01, 95% CI: -0.09, -0.01). The indirect effects assuming only one mediator were not significant (self-efficacy as the sole mediator: θ = 0.02, *SE* = 0.03, 95% CI: -0.03, -0.09; MVPA as the sole mediator: θ = -0.04, *SE* = 0.05, 95% CI: -0.16, 0.05). Total effects of the group assignment on body fat (T3) were significant, -0.96, *SE* = 0.29 (95% CI: -1.54, -0.40), with a direct effect value of -0.87, *SE* = 0.29, (95% CI: -1.44, -0.30).

The second sequential mediation model (Fig 3) was designed to test the mediating effects of the use of planning (T2) and MVPA (T3) in the relationship between the group assignment (T1) and adolescents’ body fat (T3). The analyses were conducted with two study groups including planning component (i.e., the planning group and the self-efficacy + planning group) coded as 1 and the control group coded as 0 (*n* = 906). We controlled for MVPA (T1), body fat (T1), planning (T1), age, and gender. The results of sequential mediation showed that the association between the group assignment and body fat (T3) was mediated by planning (T2) and MVPA (T3) as indicated by a significant indirect effect (θ = -0.02, *SE* = 0.01, 95% CI: -0.05, -0.01). The indirect effects assuming only one mediator were not significant (planning as the sole mediator: θ = -0.04, *SE* = 0.03, 95% CI: -0.01, -0.12; MVPA as the sole mediator: θ = -0.07, *SE* = 0.05, 95% CI: -0.19, 0.01). Total effects of the group assignment on body fat (T3) were significant, -0.91, *SE* = 0.29, (95% CI: -1.50, -0.34), with the direct effect value of -0.83, *SE* = 0.29, (95% CI: -1.41, -0.25).

Additional analyses were conducted to test two alternative models assuming that (1) the effects of the self-efficacy intervention may be mediated by T2 planning and MVPA at T3 and that (2) the effects of the planning intervention may be mediated by T2 self-efficacy and MVPA at T3. The two alternative mediator models were identical with the hypothesized mediator models (Fig 2 and Fig 3), with only one difference, i.e., the first mediator. The analyses indicated no indirect effects of the alternative mediator (i.e., planning) for the self-efficacy intervention (θ = -0.01, *SE* = 0.01, 95% CI: -0.01, 0.01) and no indirect effects of the alternative mediator (i.e. self-efficacy) for the planning intervention (θ = -0.01, *SE* = 0.02, 95% CI: -0.04, 0.04). Furthermore, secondary analyses conducted for the hypothesized models after excluding nine participants with underweight indicated significant indirect effects similar to those found in the sequential mediation analysis for the total sample (for groups including self-efficacy component, θ = -0.03, *SE* = 0.02, 95% CI: -0.07, -0.01; for groups including planning component, θ = -0.03, *SE* = 0.02, 95% CI: -0.08, -0.01).

### Moderating Effect of Built PA Facilities

To test if built PA facilities had a moderating effect we conducted a moderated mediation analysis. The two sequential mediation analyses were repeated, assuming that the indirect and direct effects are moderated by the presence of built facilities. Results for the moderating analysis in the model with self-efficacy as the mediator revealed no direct effect of built facility on body fat at T3, *B* = -1.67, *SE* = 1.11, 95% CI: -3.85, 0.52. Next, we found that the indirect effects of the group assignment on body fat (T3) were not moderated by the presence of built facilities. This was indicated by values of conditional indirect effects of self-efficacy (built facility present: θ = 0.08, *SE* = 0.07, 95% CI: -0.02, 0.28; built facility absent, θ = -0.01, *SE* = 0.03, 95% CI: -0.08, 0.04), and MVPA (built facility present: θ = -0.07, *SE* = 0.09, 95% CI: -0.28, 0.07; built facility absent: θ = -0.10, *SE* = 0.08, 95% CI: -0.29, 0.03). However, the direct effect of the group assignment on body fat (T3) was moderated by the presence of built facilities. In particular, the conditional direct effect was not significant in the absence of built facility θ = -0.47, *SE* = 0.38,95% CI: -1.22, 0.27, but it was significant when built facility was present, θ = -1.50, *SE* = 0.45, 95% CI: -2.38, -0.62.

A similar pattern of findings was observed when the moderating effects were investigated in the model with planning (T2) as the mediator. Results showed no direct effect of built facilities on body fat at T3, *B* = -1.68, *SE* = 1.02, 95% CI: -3.68, 0.32. The indirect effects of the group assignment on body fat (T3) were not moderated by the presence of built facilities. This was indicated by the non-significant values of conditional indirect effects of planning (built facility present: θ = 0.09, *SE* = 0.08, 95% CI: -0.01, 0.31; built facility absent, θ = 0.01, *SE* = 0.03, 95% CI: -0.02, 0.11), and MVPA (built facility present: θ = -0.07, *SE* = 0.07, 95% CI: -0.28, 0.02; built facility absent: θ = -0.11, *SE* = 0.07, 95% CI: -0.30, 0.01). However, the direct effect of the group assignment on body fat (T3) was moderated by the presence of built facilities for MVPA. The conditional direct effect was not significant in the absence of built facilities θ = -0.31, *SE* = 0.38, 95% CI: -1.06, 0.44, whereas it was significant when built facilities were present, θ = -1.56, SE = 0.45, 95% CI: -2.45, -0.67.

## Discussion

This longitudinal study offers an insight into the effects and mechanisms of three brief interventions using two different sets of behavior change techniques, and their combination, designed to control an increase of body fat in adolescents. Overall, the effects of the interventions on body fat were small. In general, an increase of body fat of 0.6% to 0.8% was observed from T1 to T3 (see Table 2) in intervention participants, but the increase in the control group was 1.6%. The difference between intervention and control groups is substantial and similar to 1% of body fat changes found in other PA and nutrition interventions delivered to overweight individuals [19].

One of the key issues addressed in this study refers to the potential superiority of combining two sets of intervention techniques targeting two social cognitive predictors of physical activity (self-efficacy and planning) over interventions addressing only one set of techniques (either self-efficacy or planning). Our findings indicate that in terms of weight-related outcomes at follow-up, the effects of the combined components were equal to those of the individual components alone. The similarity in the effects may be attributable to aspects of interventions that were common to each of the intervention groups [24]. These aspects could include, but are not limited to the intervention format, method of delivery, length of the intervention, method of implementation, skills of the implementers, and the support offered in the intervention setting. While a strength of the current study was to hold these aspects constant across intervention conditions so that the groups differed only in the sets of behavior change techniques relevant to each condition and addressed psychological constructs (i.e., presence or absence of self-efficacy or planning), it is possible that the presence of other very similar aspects across the conditions may have ‘washed out’ any potential for differences attributable to the intervention techniques.

In line with good practice guidelines for behavior change interventions [20], we tested potential mediators of the intervention effects. Our findings suggest that the effects of interventions may be explained by the appropriate underlying mechanisms, which include processes of fostering self-efficacy or the use of planning (for similar patterns see [18]). So far, only one study tested the mediating mechanisms of planning + self-efficacy intervention [22]. Our research and the study by Guillaumie et al. [22] used different behavior change techniques to enhance self-efficacy. This difference may explain why the two studies yielded different effects of the mediator (self-efficacy beliefs). In particular, behavior change techniques referring to mastery experience were absent in the study by Guillaumie et al. [22]. Mastery experience and self-persuasion may be among the most important sources of self-efficacy for PA [7].

The direct effects of the interventions on body fat (T3) depended to the presence of newly built PA facilities in the school environment. The findings are in line with good practice recommendations which suggest that behavior change interventions should account for changes in individuals’ cognition but also in the individuals’ physical environment [24]. However, the results indicate that built environment did not operate through perceptions of self-efficacy or the use of planning (as the indirect effects of the group assignment on body fat at T3 were not moderated by built environment). Future research should measure and analyze alternative indirect pathways through which the built environment affects body fat gain. Such pathways may account for perceived accessibility or feasibility of the environmental structures, their perceived attractiveness and features that encourage sport and fitness [3, 27].

There are several limitations of this study. Although this randomized controlled trial had a longitudinal design, the main and secondary outcomes were measured only once following the intervention. Evaluating body composition at T2 would additionally allow to estimate short-term effects of the intervention. Besides collecting post-study unstandardized reports from experimenters, we did not conduct a detailed measurement and analysis of adherence to protocol and its feasibility. Future research needs to investigate the feasibility factors which may explain implementation and delivery processes. The moderating role of the built PA facility should be generalized with extreme caution as our measure of differences in built environment referred to only one type of facility. The design of the study does not allow for concluding how the characteristics of the built environment influenced the study outcomes. The measurement of MVPA was based on self-reports whereas accelerometry would offer higher validity.

In conclusion, we found that self-efficacy, planning, and self-efficacy + planning interventions had effects on body fat that were similar in size relative to the control group. The effects on adolescents’ body fat (and MVPA) measured at 1-year follow-up were small. The interventions had preventive effects on body fat gain and the findings may be translated to approximately 1% of the difference between intervention and control groups. The effects of the interventions on body fat may be attributed to the changes in underlying psychological constructs and MVPA. Finally, the interventions had direct effects on body fat only if a built PA facilities (team sports such as soccer, basketball) were present in the near-school environment.

## Supporting Information

S1 File CONSORT Checklist, TIDieR Checklist, and Additional Supplementary Material (the Protocol). (DOC)

## References

[1] World Health Organization. Global strategy on diet, physical activity and health [Internet]. WHO.int [cited 2016 Oct 17]. Available: http://www.who.int/dietphysicalactivity/strategy/en/

[2] Ramires VV, Dumith SC, Gonçalves H. Longitudinal Association Between Physical Activity and Body Fat During Adolescence: A Systematic Review. J Phys Act Health. 2015 9;12(9):1344–58. 10.1123/jpah.2014-0222 25409296

[3] Safron M, Cislak A, Gaspar T, Luszczynska A. Effects of school-based interventions targeting obesity-related behaviors and body weight change: a systematic umbrella review. Behav Med. 2011 1;37(1):15–25. 10.1080/08964289.2010.543194 21347906

[4] Rothman KJ. BMI-related errors in the measurement of obesity. Int J Obes(Lond). 2008; 32(3 Suppl):S56–9. 10.1038/ijo.2008.87 18695655

[5] Moliner-Urdiales D, Ruiz JR, Ortega FB, Rey-Lopez JP, Vincente-Rodriguez G, España-Romero V, et al Association of objectively assessed physical activity with total and central body fat in Spanish adolescents; the HELENA Study. Int J Obes (Lond). 2009 10;33(10):1126–35. 10.1038/ijo.2009.139 19597518

[6] Schwarzer R. Modeling Health Behavior Change: How to Predict and Modify the Adoption and Maintenance of Health Behaviors. Applied Psychology. 2008 1;57(1):1–29. 10.1111/j.1464-0597.2007.00325.x

[7] Warner LM, Schüz B, Wolff JK, Parschau L, Wurm S, Schwarzer R. Sources of self-efficacy for physical activity. Health Psychol. 2014 11;33(11):1298–308. 10.1037/hea0000085 24707842

[8] Craggs C, Corder K, van Sluijs EM, Griffin SJ. Determinants of change in physical activity in children and adolescents: a systematic review. Am J Prev Med. 2011 6;40(6):645–58. 10.1016/j.amepre.2011.02.025 21565658 PMC3100507

[9] French DP, Olander EK, Chisholm A, Mc Sharry J. Which behaviour change techniques are most effective at increasing older adults’ self-efficacy and physical activity behaviour? A systematic review. Ann Behav Med. 2014 10;48(2):225–34. 10.1007/s12160-014-9593-z 24648017

[10] Olson EA, McAuley E. Impact of a brief intervention on self-regulation, self-efficacy and physical activity in older adults with type 2 diabetes. J Behav Med. 2015 12;38(6):886–98. 10.1007/s10865-015-9660-3 26162648 PMC4628895

[11] Gollwitzer PM, Sheeran P. Implementation intentions and goal achievement: A meta-analysis of effects and processes. Adv Exp Soc Psychol. 2006;38:69–119. 10.1013/S0065-2601(06)38002-1

[12] Bélanger—Gravel A, Godin G, Amireault S. A meta-analytic review of the effect of implementation intentions on physical activity. Health Psychol Rev. 2013 3;7(1):23–54. 10.1080/17437199.2011.560095

[13] Hagger MS, Luszczynska A. Implementation intention and action planning interventions in health contexts: state of the research and proposals for the way forward. Appl Psychol Health Well Being. 2014 3;6(1):1–47. 10.1111/aphw.12017 24591064

[14] Sniehotta FF, Schwarzer R, Scholz U, Schüz B. Action planning and coping planning for long-term lifestyle change: theory and assessment. Eur J Soc Psychol. 2005 7;35(4):565–576. 10.1002/ejsp.258

[15] Carraro N, Gaudreau P. Spontaneous and experimentally induced action planning and coping planning for physical activity: A meta-analysis. Psychol Sport Exerc. 2013 3;14(2):228–248. 10.1016/j.psychsport.2012.10.004

[16] Luszczynska A, Schwarzer R, Lippke S, Mazurkiewicz M. Self-efficacy as a moderator of the planning-behaviour relationship in interventions designed to promote physical activity. Psychol Health. 2011 2;26(2):151–66. 10.1080/08870446.2011.531571 21318927

[17] Schwarzer R, Luszczynska A, Ziegelmann JP, Scholz U, Lippke S. Social-cognitive predictors of physical exercise adherence: three longitudinal studies in rehabilitation. Health Psychol. 2008 1;27(1 Suppl):S54–63. 10.1037/0278-6133.27.1(Suppl.).S54 18248106

[18] Luszczynska A, Horodyska K, Zarychta K, Liszewska N, Knoll N, Scholz U. Planning and self-efficacy interventions encouraging replacing energy-dense foods intake with fruit and vegetable: A longitudinal experimental study. Psychol Health. 2016;31(1):40–64. 10.1080/08870446.2015.1070156 26160226

[19] Teixeira PJ, Carraça EV, Marques MM, Rutter H, Oppert JM, De Bourdeaudhuij I, et al Successful behavior change in obesity interventions in adults: a systematic review of self-regulation mediators. BMC Med. 2015 4;13:84 10.1186/s12916-015-0323-6 25907778 PMC4408562

[20] Abraham C, Johnson BT, de Bruin M, Luszczynska A. Enhancing reporting of behavior change intervention evaluations. J Acquir Immune Defic Syndr. 2014 8;66(3 Suppl):S293–9. 10.1097/QAI.0000000000000231 25007199

[21] Conner M, Higgins AR. Long-term effects of implementation intentions on prevention of smoking uptake among adolescents: a cluster randomized controlled trial. Health Psychol. 2010 9;29(5):529–38. 10.1037/a0020317 20836608

[22] Guillaumie L, Godin G, Manderscheid JC, Spitz E, Muller L. The impact of self-efficacy and implementation intentions-based interventions on fruit and vegetable intake among adults. Psychol Health. 2012 1;27(1):30–50. 10.1080/08870446.2010.541910 21678169

[23] Guillaumie L, Godin G, Manderscheid JC, Spitz E, Muller L. Self-efficacy and implementation intentions-based interventions on fruit and vegetable intake among adults: impact at 12-month follow-up. Glob Health Promot. 2013 6;20(2 Suppl):83–7. 10.1177/1757975913483336 23678501

[24] Horodyska K, Luszczynska A, van den Berg M, Hendriksen M, Roos G, De Bourdeaudhuij I, et al Good practice characteristics of diet and physical activity interventions and policies: an umbrella review. BMC Public Health. 2015 1;15:19 10.1186/s12889-015-1354-9 25604454 PMC4306239

[25] Bandura A. Self-efficacy: The Exercise of Control 1st ed. New York, NY: W.H. Freeman and Co.; 1997.

[26] Safron M, Cislak A, Gaspar T, Luszczynska A. Micro-environmental characteristics related to body weight, diet, and physical activity of children and adolescents: a systematic umbrella review. Int J Environ Health Res. 2011 10;21(5):317–30. 10.1080/09603123.2011.552713 21547807

[27] McGrath LJ, Hopkins WG, Hinckson EA. Associations of objectively measured built-environment attributes with youth moderate-vigorous physical activity: a systematic review and meta-analysis. Sports Med. 2015 6;45(6):841–65. 10.1007/s40279-015-0301-3 25618013

[28] Oreskovic NM, Perrin JM, Robinson AI, Locascio JJ, Blossom J, Chen ML, et al Adolescents’ use of the built environment for physical activity. BMC Public Health. 2015 3;15:251 10.1186/s12889-015-1596-6 25880654 PMC4369364

[29] Cerin E, Vandelanotte C, Leslie E, Merom D. Recreational facilities and leisure-time physical activity: An analysis of moderators and self-efficacy as a mediator. Health Psychol. 2008 3;27(2 Suppl):S126–35. 10.1037/0278-6133.27.2(Suppl.).S126 18377154

[30] Cole TJ, Bellizzi MC, Flegal KM, Dietz WH. Establishing a standard definition for child overweight and obesity worldwide: international survey. BMJ. 2000 5;320(7244):1240–3. 10.1136/bmj.320.7244.1240 10797032 PMC27365

[31] Kyle UG, Bosaeus I, De Lorenzo AD, Deurenberg P, Elia M, Manuel Gómez J, et al Bioelectrical impedance analysis-part II: utilization in clinical practice. Clin Nutr. 2004 12;23(6):1430–53. 10.1016/j.clnu.2004.09.012 15556267

[32] Cleary J, Daniells S, Okely AD, Batterham M, Nicholls J. Predictive validity of four bioelectrical impedance equations in determining percent fat mass in overweight and obese children. J Am Diet Assoc. 2008 1;108(1):136–9. 10.1016/j.jada.2007.10.004 18156000

[33] Godin G, Shephard RJ. A simple method to assess exercise behavior in the community. Can J Appl Sport Sci. 1985 9;10(3):141–6. 4053261

[34] Demographic yearbook of Poland, year 2014 [Internet]. stat.gov.pl. [cited 2015 Nov 20]. Available: http://stat.gov.pl/obszary-tematyczne/roczniki-statystyczne/roczniki-statystyczne/rocznik-demograficzny-2014,3,8.html

[35] Michie S, Ashford S, Sniehotta FF, Dombrowski SU, Bishop A, French DP. A refined taxonomy of behaviour change techniques to help people change their physical activity and healthy eating behaviours: the CALO-RE taxonomy. Psychol Health. 2011 11;26(11):1479–98. 10.1080/08870446.2010.540664 21678185

[36] Hayes AF. Introduction to mediation, moderation, and conditional process analysis: A regression-based approach 1st ed. New York: Guilford Press; 2013.

