# Supplementary material for: Self-Efficacy, Planning, or a Combination of Both? A Longitudinal Experimental Study Comparing Effects of Three Interventions on Adolescents’ Body Fat
Source: PLoS One. 2016 Jul 13;11(7):e0159125. doi: 10.1371/journal.pone.0159125 (PMC4943603; doi:10.1371/journal.pone.0159125)
Supplement: S1 File — (DOC) [file pone.0159125.s001.doc]

Supporting Information 1

CONSORT 2010 checklist of information to include when reporting a randomised trial

| Section/Topic | Item No | Checklist item | Reported on page No |
| --- | --- | --- | --- |
| Title and abstract | | | |
|  | 1a | Identification as a randomised trial in the title | 1 |
|  | 1b | Structured summary of trial design, methods, results, and conclusions (for specific guidance see CONSORT for abstracts) | 3 |
| Introduction | | | |
| Background and objectives | 2a | Scientific background and explanation of rationale | 5-10 |
|  | 2b | Specific objectives or hypotheses | 9-10 |
| Methods | | | |
| Trial design | 3a | Description of trial design (such as parallel, factorial) including allocation ratio | 10-11, 15-17 |
|  | 3b | Important changes to methods after trial commencement (such as eligibility criteria), with reasons | 15, |
| Participants | 4a | Eligibility criteria for participants | 10-11 |
|  | 4b | Settings and locations where the data were collected | 15-16 |
| Interventions | 5 | The interventions for each group with sufficient details to allow replication, including how and when they were actually administered | 15-17 and Additional Supplementary Material (Protocol) |
| Outcomes | 6a | Completely defined pre-specified primary and secondary outcome measures, including how and when they were assessed | 9-10, 11-13, 15-17 |
|  | 6b | Any changes to trial outcomes after the trial commenced, with reasons | 17 |
| Sample size | 7a | How sample size was determined | 18 |
|  | 7b | When applicable, explanation of any interim analyses and stopping guidelines | - |
| Randomisation: |  |  |  |
| Sequence generation | 8a | Method used to generate the random allocation sequence | 10-11 |
|  | 8b | Type of randomisation; details of any restriction (such as blocking and block size) | 10-11 |
| Allocation concealment mechanism | 9 | Mechanism used to implement the random allocation sequence (such as sequentially numbered containers), describing any steps taken to conceal the sequence until interventions were assigned | 10-11 |
| Implementation | 10 | Who generated the random allocation sequence, who enrolled participants, and who assigned participants to interventions | 10-11 |
| Blinding | 11a | If done, who was blinded after assignment to interventions (for example, participants, care providers, those assessing outcomes) and how | 16-17 |
|  | 11b | If relevant, description of the similarity of interventions | Additional Supplementary Material (Protocol) |
| Statistical methods | 12a | Statistical methods used to compare groups for primary and secondary outcomes | 17-18 |
|  | 12b | Methods for additional analyses, such as subgroup analyses and adjusted analyses | 17-18 |
| Results | | | |
| Participant flow (a diagram is strongly recommended) | 13a | For each group, the numbers of participants who were randomly assigned, received intended treatment, and were analysed for the primary outcome | 11 |
|  | 13b | For each group, losses and exclusions after randomisation, together with reasons | 20 |
| Recruitment | 14a | Dates defining the periods of recruitment and follow-up | 15 |
|  | 14b | Why the trial ended or was stopped | - |
| Baseline data | 15 | A table showing baseline demographic and clinical characteristics for each group | 10-11 |
| Numbers analysed | 16 | For each group, number of participants (denominator) included in each analysis and whether the analysis was by original assigned groups | 10-11 |
| Outcomes and estimation | 17a | For each primary and secondary outcome, results for each group, and the estimated effect size and its precision (such as 95% confidence interval) | 20-26 |
|  | 17b | For binary outcomes, presentation of both absolute and relative effect sizes is recommended | - |
| Ancillary analyses | 18 | Results of any other analyses performed, including subgroup analyses and adjusted analyses, distinguishing pre-specified from exploratory | 21 |
| Harms | 19 | All important harms or unintended effects in each group (for specific guidance see CONSORT for harms) | - |
| Discussion | | | |
| Limitations | 20 | Trial limitations, addressing sources of potential bias, imprecision, and, if relevant, multiplicity of analyses | 28-29 |
| Generalisability | 21 | Generalisability (external validity, applicability) of the trial findings | 26-29 |
| Interpretation | 22 | Interpretation consistent with results, balancing benefits and harms, and considering other relevant evidence | 26-29 |
| Other information | | |  |
| Registration | 23 | Registration number and name of trial registry | 17 |
| Protocol | 24 | Where the full trial protocol can be accessed, if available | 17 |
| Funding | 25 | Sources of funding and other support (such as supply of drugs), role of funders | 2 |

*We strongly recommend reading this statement in conjunction with the CONSORT 2010 Explanation and Elaboration for important clarifications on all the items. If relevant, we also recommend reading CONSORT extensions for cluster randomised trials, non-inferiority and equivalence trials, non-pharmacological treatments, herbal interventions, and pragmatic trials. Additional extensions are forthcoming: for those and for up to date references relevant to this checklist, see [www.consort-statement.org](http://www.consort-statement.org/).

Supporting Information 2

**The TIDieR (Template for Intervention Description and Replication) Checklist:**

| **Item number** | **Item** | **Where located **** | |
| --- | --- | --- | --- |
|  |  | Primary paper  (page or appendix  number) | Other ^†^ (details) |
|  | **BRIEF NAME** |  |  |
| **1.** | Provide the name or a phrase that describes the intervention. | pages 7-8 | appendix S3 |
|  | **WHY** |  | - |
| **2.** | Describe any rationale, theory, or goal of the elements essential to the intervention. | pages 3-8 |  |
|  | **WHAT** |  |  |
| **3.** | Materials: Describe any physical or informational materials used in the intervention, including those provided to participants or used in intervention delivery or in training of intervention providers. Provide information on where the materials can be accessed (e.g. online appendix, URL). | appendix S3 |  |
| **4.** | Procedures: Describe each of the procedures, activities, and/or processes used in the intervention, including any enabling or support activities. | appendix S3 |  |
|  | **WHO PROVIDED** |  |  |
| **5.** | For each category of intervention provider (e.g. psychologist, nursing assistant), describe their expertise, background and any specific training given. | appendix S3 |  |
|  | **HOW** |  |  |
| **6.** | Describe the modes of delivery (e.g. face-to-face or by some other mechanism, such as internet or telephone) of the intervention and whether it was provided individually or in a group. | appendix S3 |  |
|  | **WHERE** |  |  |
| **7.** | Describe the type(s) of location(s) where the intervention occurred, including any necessary infrastructure or relevant features. | page 12-14 |  |
|  | **WHEN and HOW MUCH** |  |  |
| **8.** | Describe the number of times the intervention was delivered and over what period of time including the number of sessions, their schedule, and their duration, intensity or dose. | appendix S3 |  |
|  | **TAILORING** |  |  |
| **9.** | If the intervention was planned to be personalised, titrated or adapted, then describe what, why, when, and how. | appendix S3 (indicated that there was no tailoring) |  |
|  | **MODIFICATIONS** |  |  |
| **10.^ǂ^** | If the intervention was modified during the course of the study, describe the changes (what, why, when, and how). | appendix S3 (indicated that there were no modifications to the intervention protocol) |  |
|  | **HOW WELL** |  |  |
| **11.** | Planned: If intervention adherence or fidelity was assessed, describe how and by whom, and if any strategies were used to maintain or improve fidelity, describe them. | N/A |  |
| **12.^ǂ^** | Actual: If intervention adherence or fidelity was assessed, describe the extent to which the intervention was delivered as planned. | appendix S3 |  |

** **Authors** - use N/A if an item is not applicable for the intervention being described. **Reviewers** – use ‘?’ if information about the element is not reported/not sufficiently reported.

† If the information is not provided in the primary paper, give details of where this information is available. This may include locations such as a published protocol or other published papers (provide citation details) or a website (provide the URL).

ǂ If completing the TIDieR checklist for a protocol, these items are not relevant to the protocol and cannot be described until the study is complete.

**Supporting Information 3**

**The content of the protocol in three experimental groups and the control group**

The procedures applied in the four study groups included pre-manipulation education, individual and group components. All procedures were delivered at schools. There were no changes to the intervention and control group procedures after the study commencement.

**Pre-manipulation education.** Across the study groups, participants received a common healthy lifestyle education program, focusing on healthy nutrition and PA, which was a part of the school curriculum. The healthy lifestyle education program lasted 8 hours. The combination of nutrition and PA interventions is in line with best practice guidelines for interventions promoting healthy body weight [1]. The education program was delivered by teachers and a group format was used. The groups discussed food composition, safe food handling, food labeling, nutrient needs for age and gender groups, dietary guidelines, and clinical nutrition issues. The program did not include behavior change techniques (BCT; [2]) and was not accompanied by changes in policies.

**General format and experimental materials.** The intervention conditions were delivered via a combination of printed forms with paper-and-pencil exercises and face-to-face sessions. All experimental conditions included an initial session (completing the forms individually in the groups + face-to-face component) and sets of handouts for three following weeks. The face-to-face component was delivered within three days of completing the initial forms. The initial session was followed by a booster session (group + face-to-face components), delivered at T2. Across the groups, completing the forms (individual component) took approximately 30 minutes and was conducted in classrooms. The face-to-face components took 45-60 minutes and were conducted in the offices of school nurses or school psychologists. The acceptability of similar protocol was evaluated earlier cf. [3]. The intervention procedures (referring to self-efficacy and planning manipulation) were not tailored to participants’ characteristics.

**Group intervention component.** At T1 participants completed the intervention materials individually, using carbonless copy paper, allowing to duplicate the forms which were filled by the students. The copies were collected for fidelity analysis and originals were left for participants. The paper-and-pencil materials followed a similar format in four groups in terms of word count, visual format, and the number and length of sections requiring participants filling in the blanks. Two independent researchers found no differences in the formal aspects, Cohen’s κs ≥ .78, *p* < .001.

**Face-to-face intervention component.** During the face-to-face component (at T1 and during the booster sessions) all participants received feedback on their body weight, information regarding their PA levels and energy expenditure based on student’s age, gender, body weight, followed by MVPA recommendations (BMI measured during the booster session was not recorded). Next, all participants and experimenters jointly reviewed the forms completed during the group component. Students were asked to report what they included in the forms and were prompted to provide detailed responses to questions included in the forms. Experimenters asked participants to read the content of the form loudly; looked for sections which were incomplete and encouraged students to complete these sections; prompted participants to provide detailed responses.

**Active control condition.** In the group component, participants were asked to read the materials and fill in the forms provided. Participants received a set of educational materials about types of physical activity (PA), PA intensity, exercise calorie expenditure, myths about PA, strength and endurance training, stretching, and general healthy nutrition guidelines. The materials excluded planning and self-efficacy statements. After reading the materials, students were asked to fill in the forms: they were asked to write down examples of their preferred type and intensity of PA mentioned in the materials, fill in a crossword on moderate-to-vigorous physical activity (MVPA), and report the most interesting information found in the materials. The forms were discussed with the experimenter. Weekly forms including instructions to write down their preferred MVPA and new things that participants learned about sport or PA for the three weeks after T1 were handed out (for similar procedure see [3]).

**Planning condition.** In the group component, participants were asked to read the materials and fill in the forms provided. The introductory part included an abbreviated version of the education materials used in the control group. The planning materials and forms had four sections: (a) information on the importance of planning, including examples of how planning works and what it affects, (b) instructions of what should be included in a good plan (the when, where, and how components), (c) formulating action and coping plans cf. [4]. The materials ended with instructions for the following 7 days to recollect/redo plans every morning.

In the planning forms participants were invited to make a plan about their MVPA for the following week, saying: ‘Using this form, make an exact plan for your MVPA, including ‘where’, ‘when’ and ‘how’. The planning form invited participants to write plans regarding MVPA. The form included a prompt for each category of the following form: ‘This is my plan about MVPA for the next 7 days. During the next week, I plan to do following exercises … (please, write down what type and intensity of exercise you plan to perform at … (write down the time), for ... (how long?), in/at … (describe the situation/place where you plan to exercise)’. Next, adolescents were prompted to form coping plans including the cues (e.g., seeing sports equipment at home, watching or reading about physically activity) and the behavioral response (engaging in MVPA). In particular, participants were asked to make coping plans regarding risky or tempting situations: ‘Throughout your day, you may be tempted to sit or lie down and not move too much. You may feel tired or busy or have no mood to exercise, it may be hot or cold outside, or you may want to play your favorite computer game for another hour. Make a plan about how you would react to these temptations of being sedentary and fill in the form’ and ‘Please try to formulate a plan which specifies your cues (e.g., a situation at home or at school or time of day or week) and your reaction (e.g., instead of playing your favorite computer game you will engage in MVPA)’. The form provided three prompts, e.g., ‘I have my own plan that will help me to maintain my regular MVPA. If I feel like watching a movie on Internet, then instead of watching it I plan … (write down what you plan to do)’. On completion of the planning forms, participants were asked to review details of their plans individually. Finally, participants were encouraged to recall (and if needed to review) their plans every morning.

During the face-to-face component weekly planning forms for the three weeks after T1 were handed out. Content of the planning forms were discussed with the experimenter.

**Self-efficacy condition.** In the group component, adolescents were asked to read the materials and fill in the forms provided. The introductory section included an abbreviated version of the educational materials used in the control group. The self-efficacy materials and forms had four sections: (a) definitions of self-efficacy beliefs, (b) information on the importance of self-efficacy for goal pursuit, (c) recollecting a mastery experience, (d) persuasive statements evoking self-persuasive statements about self-efficacy beliefs (for this protocol see also [3, 5]). This was followed by instructions for the next week: start each day with recollecting mastery experience and repeat self-efficacy statements.

In the self-efficacy forms participants were invited to read self-efficacy definitions. Participants were informed about the studies targeting self-efficacy that helped people to lead a healthy life. This part concluded that one of the ways to a healthier, physically active life is to build strong self-efficacy for dealing with barriers (e.g., temptations to engage in screen behaviors, being sedentary, feeling stressed or in bad mood). Next, participants were asked to recall an event in which they successfully acted according to their intentions and to write down their recollections e.g., referring to engaging in MVPA although they were tempted to be sedentary, or another example of performing MVPA. For example, they were asked: ‘Please try to recollect a situation when you were tempted to play a computer game or watch a movie for hours, but you engaged in physical activity instead. Or perhaps, try to recall a situation when you engaged in MVPA that you like. Try to recollect the circumstances in which this situation took place (Where was it? What type of exercise was it?)’. They were asked to write briefly about the situation (e.g., the type of exercise, where it happened, intensity and time). Further, experimenters used encouragement and persuasive statements, prompting self-efficacy, e.g.: ‘This experience shows that you can be successful and act effectively, if you only wish. You can do it! You are able to repeat this successful mastery experience in other situations, and engage in MVPA every day. Say to yourself, yes I can do it. Tell yourself that you can manage to exercise every day, even if you are tempted to do something else!’, ‘Tell yourself that you are able to change your MVPA and instead of spending more and more time sitting or lying you are able to be active’. Participants were asked to write ‘Yes, I can do it, I can do such moderate or vigorous exercises as … (examples) every day’. Next, participants were asked to recall the mastery experience and repeat self-efficacy statements every evening during the following week.

During the face-to-face component weekly self-efficacy enhancement forms for the three following weeks were handed out. Contents of the self-efficacy forms were discussed with the experimenter during the face-to-face component.

**Self-efficacy + planning condition.** This condition included all components incorporated into the self-efficacy and the planning condition. The materials and forms used in the two conditions were shortened: the number of examples was reduced and the sections to be filled in were shortened. The forms and the face-to face component was of the same length across the groups.

**Protocol adherence.** After the delivery of T2 manipulation all experimenters were interviewed about the degree in which they have followed the original protocol referring to self-efficacy and planning manipulation. The majority (11 out of 12 experimenters) indicated that the content of the educational referring to nutrition and physical activity was adjusted to participant’s knowledge. Regarding the planning and self-efficacy conditions, all experimenters indicated no major deviation in terms of the content and forms of delivery.

**Experimenters.** All experimenters (*n* = 12) had Masters’ degrees in psychology and at least one year experience in health psychology research or intervention delivery. They were responsible for enrolling the participants. Experimenters received written preparatory materials (including MVPA guidelines, energy expenditure, etc.) and participated in a 2-day training, addressing MVPA guidelines, measurement, and the study protocol. There were no changes to the intervention protocol or deviations from the planned protocol.

**References**

1. Horodyska K, Luszczynska A, van den Berg M, Hendriksen M, Roos G, De Bourdeaudhuij I, et al. Good practice characteristics of diet and physical activity interventions and policies: an umbrella review. *BMC Public Health*. 2015 Jan;15:19. doi: 10.1186/s12889-015-1354-9.
2. Michie S, Ashford S, Sniehotta FF, Dombrowski SU, Bishop A, French DP. A refined taxonomy of behaviour change techniques to help people change their physical activity and healthy eating behaviours: the CALO-RE taxonomy. *Psychol Health*. 2011 Nov;26(11):1479-98. doi: 10.1080/08870446.2010.540664.
3. Luszczynska A, Horodyska K, Zarychta K, Liszewska N, Knoll N, Scholz U. Planning and self-efficacy interventions encouraging replacing energy-dense foods intake with fruit and vegetable: A longitudinal experimental study. *Psychol Health*. 2016;31(1):40-64. doi: 10.1080/08870446.2015.1070156.
4. Luszczynska A, Sobczyk A, Abraham C. Planning to lose weight: randomized controlled trial of an implementation intention prompt to enhance weight reduction among overweight and obese women. *Health Psychol.* 2007 Jul;26(4):507-12. doi:10.1080/08870446.2015.1070156.
5. Luszczynska A, Tryburcy M, Schwarzer R. Improving fruit and vegetable consumption: a self-efficacy intervention compared with a combined self-efficacy and planning intervention. *Health Educ Res*. 2007 Oct;22(5):630-8. doi:10.1093/her/cyl133.
